# Do chain pharmacies perform better than independent pharmacies? Evidence from a standardised patient study of the management of childhood diarrhoea and suspected tuberculosis in urban India

**DOI:** 10.1136/bmjgh-2017-000457

**Published:** 2017-09-22

**Authors:** Rosalind Miller, Catherine Goodman

**Affiliations:** Department of Global Health and Development, London School of Hygiene and Tropical Medicine, London, UK

**Keywords:** health systems, tuberculosis, child health, public health

## Abstract

**Introduction:**

Pharmacies and drug stores are frequently patients’ first point of care in many low-income and middle-income countries, but their practice is often poor. Pharmacy retailing in India has traditionally been dominated by local, individually owned shops, but recent years have seen the growth of pharmacy chains. In theory, lower-powered profit incentives and self-regulation to preserve brand identity may lead to higher quality in chain stores. In practice, this has been little studied.

**Methods:**

We randomly selected a stratified sample of chain and independent pharmacies in urban Bengaluru. Standardised patients (SPs) visited pharmacies and presented a scripted case of diarrhoea for a child and suspected tuberculosis (TB). SPs were debriefed immediately after the visit using a structured questionnaire. We measured the quality of history taking, therapeutic management and advice giving against national (Government of India) and international (WHO) guidelines. We used Pearson’s χ^2^ tests to examine associations between pharmacy type and case management.

**Findings:**

Management of childhood diarrhoea and suspected TB was woefully substandard. History taking of the SP was limited; unnecessary and harmful medicines, including antibiotics, were commonly sold; and advice giving was near non-existent. The performance of chains and independent shops was strikingly similar for most areas of assessment. We observed no significant differences between the management of suspected TB in chains and independents. 43% of chains and 45% of independents managed the TB case correctly; 17% and 16% of chains and independents, respectively, sold antibiotics. We found that chains sold significantly fewer harmful antibiotics and antidiarrhoeals (35% vs 48%, p=0.029) and prescription-only medicines (37% vs 49%, p=0.048) for the patient with diarrhoea compared with independent shops. Not a single shop managed the patient with diarrhoea correctly according to guidelines.

**Conclusion:**

Our results from Bengaluru suggest that it is unlikely that chains alone can solve persisting quality challenges. However, they may offer a potential vehicle through which to deliver interventions. Future intervention research should consider recruiting chains to see whether effectiveness of interventions differ among chains compared with independents.

**Key questions**

**What is already known about this topic?**

- It is widely reported that pharmacy practice in many low-income and middle-income countries (LMICs) is substandard.
- Chain pharmacies are expanding in India and other LMICs.
- Economic theory predicts that chains may positively affect quality, but very little is known about this in practice in pharmacy markets.

**What are the new findings?**

- Quality of care for childhood diarrhoea and suspected tuberculosis, as measured by ideal case management of standardised patients and adherence to recommended history-taking and advice-giving lists, was found to be equally poor in chains and independent pharmacies in Bengaluru, India.
- Chains sold significantly fewer prescription-only medicines and those deemed ‘harmful’ for the patient with diarrhoea, compared with independent shops.

**Recommendations for policy**

- Introduction of chains alone is unlikely to address quality challenges in this context.
- Interventions to improve effectivess should be delivered through chains to see whether they yield different results compared to individual shops.

## Introduction

It has been widely established that the care received from pharmacies in many low-income and middle-income countries (LMICs) is inadequate, despite frequently being patients’ first point of contact with the healthcare system.^1–3^ All too often, medicine use in these settings is ‘irrational’. That is, patients do not receive the appropriate medicines, in doses that meet their individual requirements, for an adequate duration, and at the lowest cost.^4^ Irrational medicine use is a major public health concern, which contributes to unnecessary morbidity and mortality; fuels the growing threat of antimicrobial resistance (AMR); and leads to needless spending.^5^ AMR has garnered particular interest of late. WHO has declared it a ‘global health security emergency’, and 193 heads of states and governments have pledged to address the concern through a UN declaration.^6 7^ In approaches to prevent the situation worsening, pharmacies should arguably be a key focus.

In recent years, there has been a growing interest in pharmacy chains in LMIC contexts.^8–10^ India, in particular, has seen prolific expansion of the corporate pharmacy retail sector.^9^ In theory, there are reasons to believe that chains may positively influence key determinants of treatment behaviour, notably the regulatory environment and profit incentives faced by staff.^3^ The fragmented nature of retail pharmacy in India makes enforcing regulation costly and logistically difficult. Through consolidation, chains could improve regulation in two ways. State regulation can be concentrated on central management structures; essentially, the regulator can make firms take responsibility for local branches. Second, firms may self-regulate in order to preserve brand identity.^11^ Another reason why quality may improve in a chain situation concerns incentive structures. Financial incentives faced by pharmacy-level personnel working in chains are low powered compared with those who own their own pharmacy and directly receive the profits of transactions.^12^ Independent shopkeepers may therefore face stronger incentives to engage in irrational medicine use, such as selling unnecessary or prescription-only medicines (POMs) without a prescription, where this increases their profits.

The question of whether this organisational structure of the pharmacy firm affects quality of care remains largely untouched by empirical research studies in LMIC. Studies from high-income countries report on a wide range of outcomes,^13–15^ but given the strength of regulation in high-income contexts, the findings are likely to have limited applicability in LMICs. Two studies from Mexico report on the sale of misoprostol (to induce abortion). One found that chains were less likely to sell misoprostol compared with independent shops,^16^ while the other found no significant differences.^17^ Others examined the effect of chain store entry on prices and drug quality in the retail pharmacy market in Hyderabad, India, but did not assess therapeutic management of patients.^18^

To address this gap in the literature, this paper reports on a study from Bengaluru, South India, which used standardised patients (SPs), often considered the ‘gold standard’ for quality of care measurement,^19^ to assess the case management of two conditions, in a representative sample of chain and independent pharmacies. Symptoms of these conditions are commonly presented in pharmacies, represent a high burden of disease and are subject to key quality concerns in relation to underprovision and overprovision of treatment.

## Methods

### Study setting and design

Pharmacy chains are concentrated in populous cities, with the greatest concentration in South India. This guided the selection of Bengaluru, the capital of Karnataka State and India’s third most populous city, as the study site.

We used a cross-sectional SP survey to investigate the management of suspected tuberculosis (TB) in an adult and acute watery diarrhoea in a 2 year old (who was not present). WHO recommends non-bacterial diarrhoea in children under age 5 as a tracer condition for measuring quality of care. Other health problems that are either frequently presented or of particular clinical or economic importance are also deemed appropriate provided that there are clear treatment guidelines.^20 21^ In India, TB meets these criteria.^22 23^ These conditions also represent contrasting examples of recommended management—pharmacies should refer a suspected TB case, whereas diarrhoea is commonly wholly treatable in the pharmacy environment.

We trained six research assistants (one female and five males) as SPs to visit chain and independent pharmacies. SPs were recruited from Bengaluru where the study was undertaken. The average age was 31, the youngest being 20 and the oldest 45. The individuals were trained (in a group) by RM (with input from a local researcher) over a period of 1 week. Training included learning the cases, including much role play, and running through the debrief questionnaire in the classroom. All SPs also ran through the details of their back-story and practised answering questions relating to their personal situation, as well as the presentation of the medical case. Each SP completed a number of pilot presentations in the field, under the supervision of a senior research assistant, until we were confident that the cases were being presented in a standardised, convincing manner. It was discussed among the research team how they should dress to ensure their social background appeared similar to a typical customer who might present such cases.

The SPs presented a rehearsed, scripted scenario, in Kannada (the local language), of both conditions, during a single visit, at a random sample of pharmacies. SPs purchased any recommended medications. Immediately after each pharmacy visit, they were debriefed using a structured questionnaire. We measured the quality of case management based on national and international guidelines. For the TB case, we used Government of India (GOI) and the Indian Pharmaceutical Association guidelines for community pharmacists.^23^ In the absence of any pharmacy-specific Indian or international guidelines for the management of diarrhoea, we based our assessment on WHO guidelines (also adopted by GOI) aimed at ‘physicians and other senior health workers’.^24 25^ These can be argued to be appropriate for pharmacy staff as India’s ‘Pharmacy Practice Regulations’ include as responsibilities of community pharmacists ‘selling over-the-counter medicines; counselling and advising the public on the treatment of minor ailments’,^26^ and the diarrhoea case presented here can be considered a minor ailment.

### Cases

Table 1 provides an overview of the cases, how the SPs presented them and the expected course of action against which we measured their management. According to the guidelines, community pharmacists should refer patients with symptoms suggestive of TB for sputum examination. They should not sell medicines that require a prescription. The sale of medicines from Indian pharmacies is governed by the Drugs and Cosmetic Rules 1945. Under this act, medicines are categorised as over-the-counter (OTC) (no schedule) or prescription-only. There are three levels of POMs: H, H1 and X. Schedule H medicines require a prescription from a qualified practitioner. Schedule H1 was introduced in 2013 to curb OTC use of certain POM medicines (mainly antibiotics). The 46 H1 medicines are subject to an extra set of conditions on dispensing (identity of the patient, contact details of the prescriber and the name and dispensed quantity of the drug must be recorded in a separate register). Schedule X is the most restrictive list, comprising a small number of narcotics, for which the pharmacy is required to retain the prescription for 2 years.

**Table 1:** Standardised patient case details and expected management

Case details		Expected management		
Case description	Details of scenario	History	Treatment	Advice
Acute watery diarrhoea in a child	“I need to buy something for my niece who has diarrhoea. She is 2 years old?” Further questioning would reveal: Four episodes during the last day; More thirsty than usual; May have had a slight fever; No blood in the stool, abdominal pain or vomiting; No medication had been taken.	Pharmacy server to ask: Blood in stool? Duration of diarrhoea? Number of stools per day? Number of episodes of vomiting? Presence of fever? Preillness feeding practices? Type of fluids and foods during illness? Child passing urine? Tried any medication?	Oral rehydration therapy using ORS solution; Zinc supplementation; No sale of antidiarrhoeals, antibiotics or antispasmodics.	Explain how to use ORS; Importance of more fluids; Usual diet should be continued (including milk); Take to health worker if signs of dehydration or other problems, for example, blood in stool.
Suspected pulmonary tuberculosis in an adult	On completion of diarrhoea advice: “Also for myself… I have had cough and some fever for 3–4 weeks. We have had a relative staying with us who has TB. Can you suggest something?” Further questioning would reveal: Sputum in the cough; Sweating at night; Loss of appetite; No medication had been taken.	Pharmacy server to ask: Consulted doctor? Chest pain? Sputum or blood in cough? Weakness or fatigue? Weight loss? Loss of appetite? Chills? Night sweats? Any other symptoms? Tried any medication?	Referral to TB clinic or other healthcare provider for sputum examination; No sale of antibiotics (including anti-TB medication) or steroids.	Advise that treatment is available free of charge from government hospitals.

ORS, oral rehydration salts; TB, tuberculosis.

We considered provision of antibiotics (including anti-TB medicines) and/or steroids as ‘harmful’, in line with Satyanarayana and colleagues; incomplete and unneeded courses of antibiotics could lead to emergence of resistant strains and steroids could mask the symptoms leading to delayed diagnosis.^27^ A 2-year-old child with diarrhoea should be managed with oral rehydration therapy (ORT), supplemental zinc and continued feeding of energy-rich foods and breast feeding. Again, POMs should not be sold, and in this case, antibiotics, antidiarrhoeals and antispasmodics are categorised as ‘harmful’.^25 28^ We term medicines not listed in the guidelines yet not deemed ‘harmful’ as ‘not recommended’. For both cases, we expect pharmacy staff to ask questions to confirm the diagnosis and determine appropriate treatment and to provide advice.

### Selection of pharmacies and sample size

We obtained a list of all pharmacies registered in the Bengaluru urban district from the Karnataka State Drug Control Department. To check the comprehensiveness and validity of this list, we completed censuses in three neighbourhoods with differing wealth profiles (according to local informants). This exercise confirmed that the list provided a comprehensive sampling frame (97% accurate). From the list, we then categorised pharmacies as either ‘independent’ or ‘chain’. We defined chains as organisations where two or more pharmacies were operating under the same name and the business used distinctive branding across all pharmacies. We excluded pharmacies operating inside hospitals or clinics, which customers could not access from the street. The resulting list contained 5135 independents and 529 chains shops deriving from 13 chains (table 2). Subsequently, we selected a random sample of pharmacies, stratified by type: chain or independent. Our sample included shops from eight chains: the largest seven and one chain of the 2–5 outlet size (details in table 2).

**Table 2:** Size of pharmacy chains in Bengaluru and in sample

Outlets (n)	Chains (n)	Chains in our sample (n)	Outlets in our sample (n)
2–5	6	1	1
6–10	2	2	4
11–50	2	2	6
51–100	1	1	14
101+	2	2	78

Between May and June 2015, SPs visited 333 pharmacies across Bengaluru (103 chains and 230 independents) and presented both cases at each pharmacy. These figures satisfy sample size calculations based on a level of significance of 0.05, 80% power, effect size of 0.2 (ie, can detect a 20% difference in quality measurements between pharmacy types) and a proportion of interest of 0.5 for the outcome of correct case management.

### Statistical analysis

We used STATA 14 to analyse the two disease cases separately. The unit of analysis was the pharmacy. We used Pearson’s χ^2^ tests to examine the effects of pharmacy type (chain vs independent) on history taking, treatment recommendations and advice.

### Ethical approval

The London School of Hygiene and Tropical Medicine Ethics Committee in London, England, and the Society of Community Health Awareness Research and Action Institutional Scientific and Ethics Committee in Bengaluru, India, approved the study. We specifically sought and received approval to waive obtaining informed consent from the pharmacies prior to the SP visits.

## Results

We present the results of the SP survey according to the three key aspects of case management specified in table 1.

### History taking

Questioning of SPs by pharmacy staff was generally poor (table 3), with no significant differences in history taking at chain and independent pharmacies. Less than 10% of chains and independents asked a single recommended question regarding the diarrhoea case. This figure was slightly higher for suspected TB with 17% of chains and 23% of independents asking at least one relevant question.

**Table 3:** History taking in chain and independent pharmacies when presented with cases of suspected TB and diarrhoea

Case	Recommended questions	Chain (n=103) % (95% CI)	Independent (n=230) % (95% CI)	p Value*
Suspected TB	Consulted a doctor?	1.9 (0.5 to 7.5)	3.5 (1.7 to 6.8)	0.448
	Chest pain?	1.0 (0.13 to 0.67)	2.6 (1.2 to 5.7)	0.336
	Sputum or blood in cough?	11.7 (6.7 to 19.5)	13.5 (9.6 to 18.6)	0.646
	Weakness or fatigue?	0	0.9 (0.2 to 3.4)	0.342
	Weight loss?	0	0	
	Loss of appetite?	0	0	
	Chills?	0	0	
	Night sweats?	1.0 (0.1 to 0. 7)	1.7 (0.7 to 4.6)	0.594
	Any other symptoms?	4.9 (2.0 to 11.2)	1.7 (0.7 to 4.6)	0.105
	Tried any medication?	0	1.7 (0.7 to 4.6)	0.178
	Asked any recommended questions?	16.5 (10.5 to 25.0)	22.6 (17.7 to 28.5)	0.204
Diarrhoea	Blood in stool?	0	0	
	Duration of diarrhoea?	3.9 (1.5 to 10.0)	4.4 (2.4 to 7.9)	0.845
	Number of stools per day?	4.9 (2.0 to 11.2)	2.2 (0.90 to 5.1)	0.185
	Number of episodes of vomiting?	1.9 (0.5 to 7.5)	3.9 (2.0 to 7.4)	0.352
	Presence of fever?	0	0.9 (0.2 to 3.4)	0.342
	Preillness feeding practices?	0	0	
	Type of fluids and foods during illness?	0	0	
	Child passing urine?	0	0.4 (0.1 to 3.1)	0.503
	Tried any medication?	0	0	
	Asked any recommended questions?	9.7 (5.3 to 17.2)	9.1 (6.0 to 13.6)	0.867

*Estimated by Pearson’s χ^2^ test.

Recommended questions for the suspected TB case are primarily to confirm the diagnosis. The most commonly asked question was regarding the presence of blood or sputum on coughing, asked by 12% of chains and 13% of independents. Less than 5% of chains and independents asked about action already taken or other questions to determine diagnosis including the presence of night sweats, pain in the chest, fatigue or any other symptoms. No shops asked about weight loss, loss of appetite or chills.

Correct questioning of the diarrhoea case would rule out a more serious underlying condition warranting referral for medical attention. No more than 5% of chains or independents asked any recommended questions. Not a single shop made enquiries regarding remedies already taken, fluid and food intake of the child either before or since falling ill or the presence of blood in the stool.

### Therapeutic management

**Figure 1:**
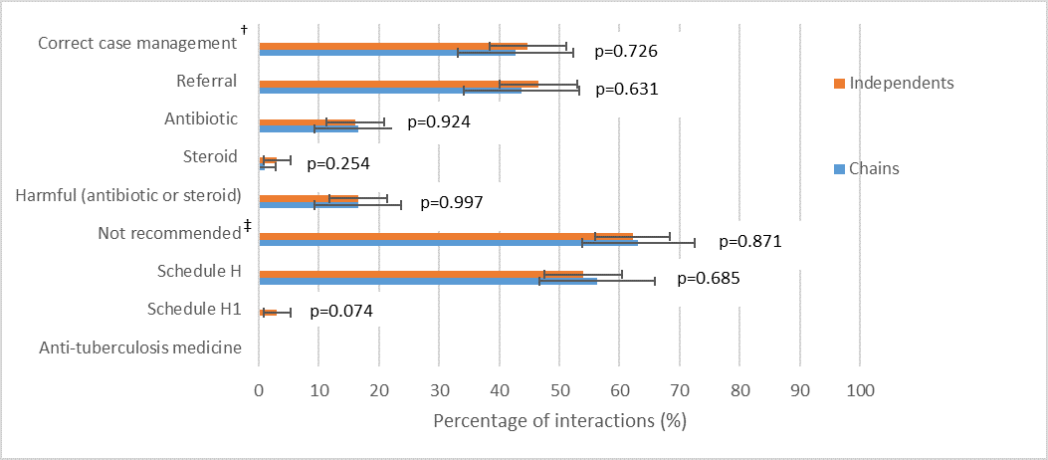
Therapeutic management of suspected TB case by independent and chain pharmacies (see online supplementary appendix A1 for corresponding table). p Values were estimated using Pearson’s χ^2^ test. †Correct case management defined as referral without sale of any ‘harmful’ medicines (antibiotic or steroids). ‡We define ‘not recommended’ medicines as those not listed in the guidelines yet not deemed ‘harmful’. They include cough and/or cold medicines, analgesics and antiacid.

**Figure 2:**
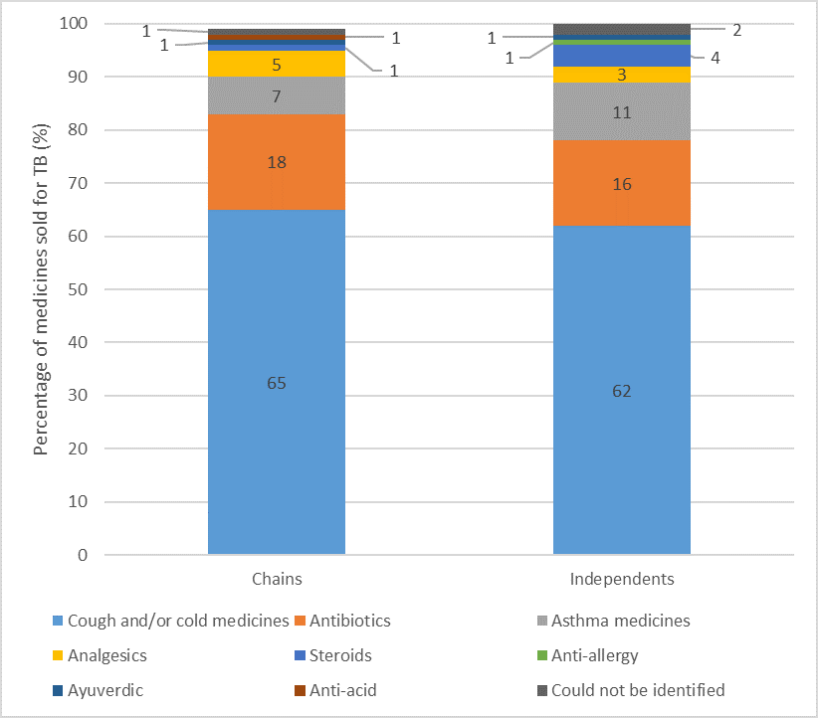
Breakdown of medicines sold for suspected TB case by outlet type. May not add to 100% due to rounding. TB, tuberculosis.

Figure 1 (and online supplementary appendix A1 for corresponding table) shows that SPs received correct management of the suspected TB case in 43% and 45% of chain and independent shops, respectively. We observed no significant differences between the therapeutic management of suspected TB in chains and independents. In terms of harmful medicines, shops rarely gave steroids, but 16% of chains and independents sold antibiotics^i^. A large proportion of chains and independents sold medicines that are not recommended for the treatment of TB: 63% and 62%, respectively. Over 50% of both shop types sold a schedule H medicine, although chains did not sell any of the more restricted H1 drugs. Seven independent shops (3%) sold H1 medicines (all of which were antibiotics). It is notable that not a single shop sold first-line anti-TB medicine. Figure 2 shows, of medicine sales, the proportion accounted for by each medicine category. The results are strikingly similar for chains and independents. Almost two-thirds of medicines sold were cold and/or cough preparations. The second and third most commonly sold medicines were antibiotics and antiasthma drugs, respectively.

10.1136/bmjgh-2017-000457.supp1 Supplementary file 1

**Figure 3:**
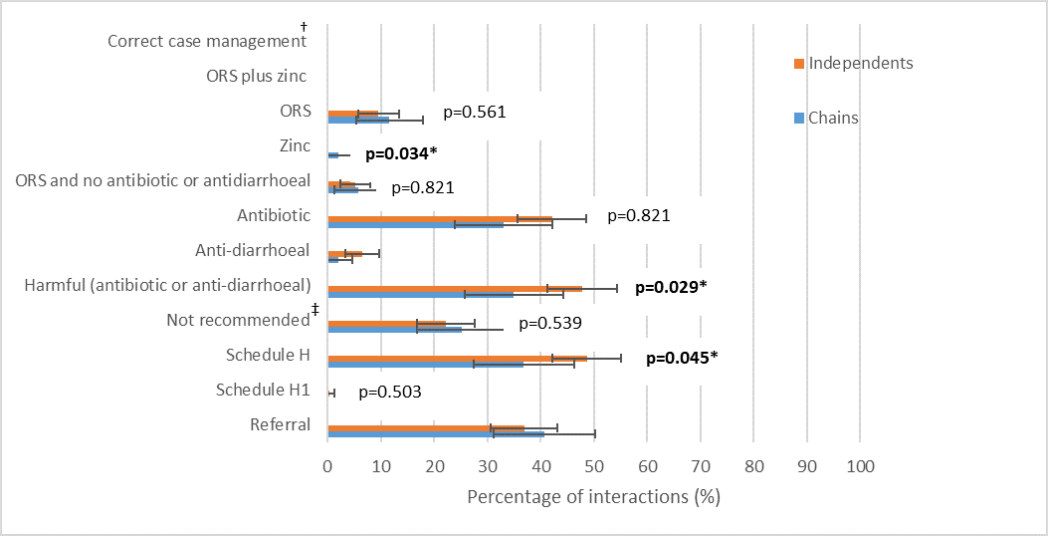
Therapeutic management of diarrhoea case by independent and chain pharmacies (see supplementary appendix A2 for corresponding table). p Values were estimated using Pearson’s χ^2^ test. †Correct case management defined as provision of ORS and zinc and no ‘harmful’ medicines (antibiotics, antidiarrhoeals or antispasmodics). ‡‘Not recommended’ medicines include prebiotics and probiotics, analgesics, antihelminitic and antiallergy. *Statistically significant at the 5% level. ORS, oral rehydration salts.

No shops managed the case of childhood diarrhoea according to current guidelines (figure 3 and online supplementary appendix A2 for corresponding table). Further, no shops sold oral rehydration salts (ORS) and zinc together. The sale of zinc was extremely rare, recommended by only two chain shops (2%) and no independents (p=0.034). Only 12% of chains and 10% of independents sold ORT and in exactly half of these sales; this was alongside harmful medicines. A total of 33% of chains and 42% of independents sold antibiotics. Antidiarrhoeal use was also higher in independents, 7% vs 2% for chains. Both antibiotics and antidiarrhoeals are considered harmful in the management of this case, and chains were found to sell significantly fewer of this combined category of harmful medicines (35% vs 48%, p=0.029). Antibiotics^ii^ or antidiarrhoeals accounted for nearly all the schedule H medicines sold; thus, we see significantly fewer chains selling schedule H medicines (37% vs 49%, p=0.045). H1 category medicine was sold by one independent pharmacy and no chains. A quarter of chains and 22% of independents sold treatments that are not recommended for diarrhoea. Although non-bacterial diarrhoea can be managed in the pharmacy, 41% of chains and 37% of independents referred the child to a doctor.

**Figure 4:**
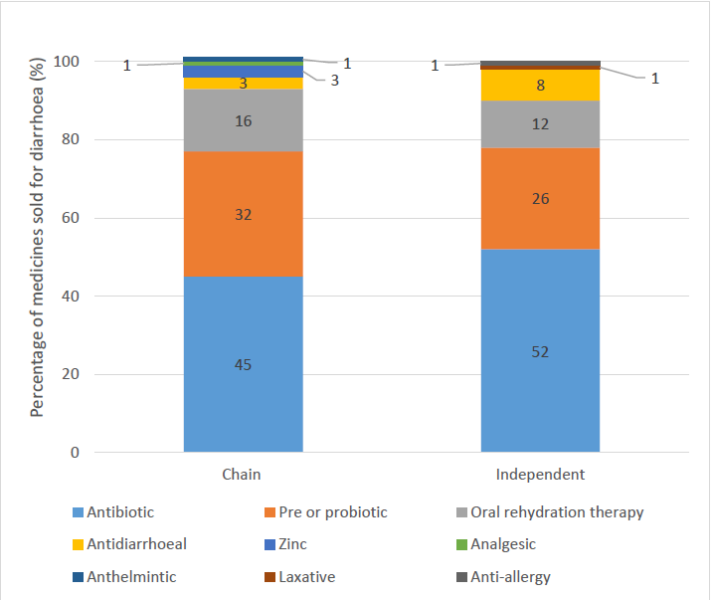
Breakdown of medicines sold for diarrhoea case by outlet type.

Of medicines sold for the patient with diarrhoea, worryingly, antibiotics made up the largest proportion for both chains and independents, 45% and 52%, respectively (figure 4). Prebiotics and probiotics were popular, accounting for 32% of sales in chains and 26% in independents. ORT only accounted for 16% and 12% of sales in chains and independents, respectively.

We also demonstrated that the findings for treatment were unaffected after adjusting for any SP-specific effects (online supplementary appendix A3, 4).

### Advice giving

Advice giving was uniformly limited. Only a handful of shops advised patients that TB treatment was available free of charge from the government (table 4). General advice for the fictitious child with diarrhoea was close to non-existent. While 41% of chains and 37% of independents referred the childhood diarrhoea case in the first instance, of the remaining shops, less than 1% of both shop types advised the SP to seek medical attention if they noticed any warning signs. One independent and no chains explained the importance of giving the child extra fluids to prevent dehydration. Advice regarding diet was similarly poor, given by 0.95% of chains and 1.75% of independents. We only observed one significant difference in terms of advice giving. Of shops that did sell ORS, chains were found to offer an explanation of how to make up and use the solution in a larger proportion of interactions, 56% vs 10% for independents. The absolute numbers that provided ORS were, however, low.

**Table 4:** Advice giving by chain and independent pharmacies

Case	Advice	Chain (n=103) % (95% CI)	Independent (n=230) % (95% CI)	p Value*
Suspected TB	Treatment available free of charge from government hospital	1.0 (0.1 to 6.7)	1.7 (0.7 to 4.6)	p=0.594
Diarrhoea	Advised to visit doctor if any warning signs†	1.0 (0.1 to 6.7)	0.9 (0.2 to 3.4)	p=0.928
	Explained importance of giving extra fluids	0	0.5 (0.1 to 3.1)	p=0.503
	Gave advice regarding diet	1.0 (0.1 to 6.7)	1.8 (0.7 to 4.6)	p=0.594
		Chain (n=9)	Independent (n=20)	
	Explain how to make up and use ORS? (of those providing)	55.6 (22.5 to 84.3)	10.0 (2.3 to 34.7)	p=0.008*

*Estimated by Pearson’s χ^2^ test.

†This indicator excludes pharmacies that referred the patient to a medical practitioner as an initial course of action (see figure 2 for these data).

ORS, oral rehydration salts.

## Discussion

To our knowledge, this is the first study to compare the quality of all aspects of case management at chain and independent pharmacies for any condition in an LMIC setting. Using SPs provides an accurate picture of how pharmacy staff manage these conditions in everyday life, and the standardised presentation allows for direct comparison across pharmacies.^29^ Our results showed that the management of both cases did not live up to national or international standards, in either chains or independents. In terms of history taking, there were no significant differences between pharmacy types for either condition, and the level of questioning by pharmacy staff would not have elicited the required information to manage the SPs appropriately. We observed no significant differences in therapeutic management of the patient with suspected TB. Fewer than half of both shop types managed the case correctly by referring without selling any harmful medicines. The one other SP study that has investigated how pharmacies respond to TB in India showed that antibiotic use substantially decreased when SPs presented with a known diagnosis, as opposed to just symptoms.^27^ In our scenario, while SPs did not present with a medically confirmed diagnosis, they mentioned contact with an infected individual and hence a suggestion that they might have TB. This is more in line with a known diagnosis and our results are therefore likely presenting pharmacy behaviour at the more positive end of the spectrum. As with all SP studies, our results reflect how pharmacies manage an unknown individual, and we cannot be sure this is the same as for regular customers.

Therapeutic management of the diarrhoea case was even worse than that of suspected TB. No pharmacies managed the case correctly. Chains did, however, sell significantly more zinc and fewer medicines categorised as harmful. This translated into chains selling significantly fewer schedule H medicines. Advice giving was almost non-existent in both types of pharmacies. The lower sales of POMs for the patient with diarrhoea translated into significantly reduced cost of the diarrhoea consultation in chains compared with independent shops (draft in preparation). Further, given that patients are paying for their medicines out of pocket when seeking care at the pharmacy, we must note the unnecessary spending on both ‘not recommended’ and ‘harmful’ medicines.

The use of H1 medicines was scarce for both cases, but it is worth highlighting that not a single chain sold a medicine of this category (compared with eight independents). This is an important finding, which corroborates the research of Satyanarayana and colleagues who report that pharmacies in other Indian cities (Mumbai, Patna and Delhi) also did not sell any first-line anti-TB medicines when presented with an SP.^27^ Adherence to restrictions on the prescription of H1 medicines appears to be a positive finding for TB control efforts.

This study also brings to our attention the lack of guidance for the treatment of minor ailments in the Indian (and other LMIC) pharmacy setting and highlights the grey area of how the management of conditions such as diarrhoea should be assessed. Considering the high proportion of childhood deaths accounted for by diarrhoea in India and the high utilisation of pharmacies, the development of pharmacy-specific treatment guidelines seems long overdue.

We report results from one city, raising the question of whether the findings are generalisable to other urban centres where chains operate. While other research has not focused on chains, our results add to a growing body of research in other areas of India that has used SP to assess quality of care for both pharmacies and other primary healthcare providers, including for childhood diarrhoea and TB. These other studies have shown quality of case management to be ubiquitously poor, regardless of whether patient first contact is with a pharmacy, a medically qualified practitioner or an unqualified, informal provider.^27 30–32^ Many of the chains in Bengaluru also operate in other Indian cities, where they would be likely to face similar incentives. Pharmacies across India operate under the same regulatory controls, and despite potential state-level variations in regulatory implementation, regulatory failure has been reported to be widespread across the country.^33–37^ Moreover, reviews have found striking similarities in the determinants of pharmacy provider behaviour in LMIC across countries and even continents,^3 38 39^ meaning that these results are potentially applicable to other LMIC settings with similar regulatory challenges. Other LMICs that have either an established or growing corporate pharmacy retail sector include Mexico, South Africa, Nigeria, Kenya, Uganda and the Philippines.^8–10^ Further study would be worthwhile to determine whether differences exist in other settings, where the business models for chains may differ.

While we report the results from the suspected TB and diarrhoea scenarios separately, SPs presented the details of the cases in a single encounter at each pharmacy, rather than the more common approach of using separate interactions. Treatment results are in keeping with other studies that presented one of these conditions as a standalone case at pharmacies in India.^27 40^ We, therefore, have no reason to believe that pharmacy staff acted differently because SPs presented both cases in a single client interaction.

While no other studies have compared chain and independent pharmacies in terms of quality of care in an LMIC setting, Bennet and Yin examined the effect of chain store entry on prices and drug quality in Hyderabad, India.^18^ Through collaboration with a pharmacy chain, they gathered baseline data in markets the firm wished to enter and later resurveyed markets 1 year after market entry. The paper reported that, compared with independent retailers, chain prices were 6% lower and pharmacopoeia compliance (drug quality) was 6% higher. The resulting effect of chain entry on the market was a relative 5% improvement in drug quality and a 2% decrease in prices at existing retailers. If chain entry had led to similar spill over quality improvement effects in our study site, this would imply that our measured effect of a chain on quality would be biased towards zero. We do not have sufficient geographical data in order to determine whether the presence of a chain shop nearby alters the behaviour of independents, though this would be an interesting area for future research. However, a review of pharmacy practice in Asia reported similar management for various presentations of childhood diarrhoea going back 30 years, that is, before the advent of chains, indicating that major changes in the behaviour of independents do not appear to have occurred in recent times.^3^

The main difference between pharmacy types observed was the lower use by chains of harmful POMs for the patient with diarrhoea. There is evidence that some of these POMs, such as antibiotics, are high profit generators.^41–43^ Lower-powered financial incentives faced by chain staff may explain a reduction in their use. Through in-depth interviews with chain and independent staff that we completed as a complement to this SP survey, we have been able to gain a more in-depth understanding of the behaviour of chains and independents (draft in preparation). We found that chain staff were heavily incentivised through a combination of sales targets and pressure from head-office and hence the incentives they faced were, in fact, not low powered. Additionally, these interviews revealed that profit-maximising strategies of chain employees tended to focus on improving customer experience, whereas independent owners focused on medicines sales. In addition to profit concerns, knowledge and regulation have also been shown to be important determinants of pharmacy practice. Our qualitative work showed that stronger penalties for the provision of the more restricted medicine schedules have been effective and some chains have instigated processes to ensure these rules are not broken (ibid). Chains use a number of methods to self-regulate and are able to control staff practices, but their main area of concern is customer satisfaction, rather than rational medicine use. Only a minority of chains had measures to monitor the sale of POMs at their outlets. In terms of knowledge, there appears to be little difference between knowledge of those working in chain and independent pharmacies. Even were there to be differences, it is well established that there is a large gap between what providers know and what they do in India, indicating that knowledge is necessary but not sufficient to ensure best practice.^27 30–32 44^

Our results provide a starting point for investigating how the organisation of the pharmacy firm affects provider behaviour in an LMIC setting. We did find small differences between the behaviour of chain staff and independent pharmacy owners. However, chains were far from fully addressing the quality deficiencies observed in the retail pharmacy sector, and it is unlikely that chains alone are going to solve persisting quality challenges. However, they may offer a potential vehicle through which to deliver interventions. Intervention initiatives reported in the literature include training, intensification of regulatory controls, peer review and accreditation.^45–47^ Some strategies, such as peer review and performance management, may have more impact within the structure of a chain. ‘League tables’ and ‘naming and shaming’ have been used in high-income settings to influence prescribing behaviour of doctors working within a common organisation.^48^

## Conclusion

The performance of chains and independent shops was equally poor for most areas of assessment. Our results indicate that, while in theory chains have the potential to improve treatment behaviour, in practice, they are unlikely to offer a magic bullet solution. However, importantly, we found that chains sold fewer prescription-only and harmful medicines to the patient with diarrhoea and successfully self-regulated the sale of H1 medicines. Future intervention research should consider recruiting chains to see whether intervention effectiveness differs among chains compared with independents.

## Footnotes

i Top five antibiotics sold for suspected TB case: (1) amoxicillin; (2) azithromycin; (3) ciprofloxacin; (4) levofloxacin; (5) erythromycin.

ii Top five antibiotics sold for diarrhoea case: (1) metronidazole+norfloxacin; (2) ofloxacin; (3) ofloxacin+ornidazole; (4) ofloxacin+metronidazole; (5) metronidazole.
