# Supplementary material for: Do chain pharmacies perform better than independent pharmacies? Evidence from a standardised patient study of the management of childhood diarrhoea and suspected tuberculosis in urban India
Source: BMJ Glob Health. 2017 Sep 22;2(3):e000457. doi: 10.1136/bmjgh-2017-000457 (PMC5623271; doi:10.1136/bmjgh-2017-000457)
Supplement: Supplementary file 1 [file bmjgh-2017-000457supp001.pdf]

Appendix

Table A1: Therapeutic management of suspected TB in chain and independent pharmacies

Variable	Chain (n=103) % (95% CI)	Independent (n=230) % (95% CI)	P-value†
Correct case management	42.7 (33.5-52.5)	44.8 (38.4-51.3)	p=0.726
Referral	43.7 (34.4-53.5)	46.5 (40.1-53.0)	p=0.631
Antibiotic	16.5 (10.5-25.0)	16.1 (11.9-21.5)	p=0.924
Steroid	1.0 (0.1-6.7)	3.0 (1.5-6.3)	p=0.254
Harmful (antibiotic or steroid)	16.5 (10.5-25.0)	16.5 (12.2-21.9)	p=0.997
Not recommended	63.1 (53.3-71.9)	62.2 (55.7-68.2)	p=0.871
Schedule H	56.3 (46.5-65.6)	53.9 (47.4-60.3)	p=0.685
Schedule H1	0	3.0 (1.5-6.3)	p=0.074
Anti-tuberculosis medicine	0	0	

†estimated by Pearson Chi ^{2} test

Table A2: Therapeutic management of childhood diarrhoea in chain and independent pharmacies

Variable	Chain (n=103) % (95% CI)	Independent (n=230) % (95% CI)	P-value†
Correct case management	0	0	
ORS plus zinc	0	0	
ORS	11.7 (6.7-19.5)	9.6 (6.3-19.5)	p=0.561
Zinc	1.9 (0.5-7.1)	0	**p=0.034***
ORS and no AB or AD	5.8 (2.6-12.4)	5.2 (3.0-9.0)	p=0.821
Antibiotic (AB)	33.0 (24.6-41.7)	42.2 (35.9-48.7)	p=0.114
Antidiarrhoeal (AD)	1.9 (0.5-7.5)	6.5 (4.0-10.6)	p=0.079
Harmful (AB or AD)	35.0 (26.3-44.7)	47.8 (41.4-54.3)	p=**0.029***
Not recommended	25.2 (17.7-34.6)	22.2 (17.2-28.0)	p=0.539
Schedule H	36.9 (28.1-46.7)	48.7 (42.3-55.2)	p=**0.045***
Schedule H1	0	0.43 (0.1-3.0)	p=0.503
Referral	40.8 (31.7-50.6)	37.0 (30.9-43.4)	p=0.507

†estimated by Pearson Chi ^{2} test
*significant at 5% level

We used a mixed effect logistic regression model to assess the association between pharmacy type and components of therapeutic management for both tracer conditions. In this model we include standardised patient (SP) fixed effects in order to eliminate any SP-specific effects from the estimates. The adjusted odds ratios (for both the diarrhoea and suspected TB case) do not significantly differ from the unadjusted data, showing that inter-rater reliability is not what is driving our results.

Table A3: Impact of pharmacy type on therapeutic management of diarrhoea case, with and without SP fixed-effects

Variable	Unadjusted Odds Ratio	95% Lower Bound	95% Upper Bound	P-value	Adjusted Odds Ratio	95% Lower Bound	95% Upper Bound	P-value
Correct case management	n/a				n/a			
ORS plus zinc	n/a				n/a			
ORS	0.8	0.38	1.69	0.562	0.71	0.33	1.54	0.388
Zinc	1†				1			
ORS and no antibiotic or antidiarrhoeal	0.89	0.32	2.44	0.821	0.79	0.28	2.24	0.661
Antibiotic	1.48	0.91	2.41	0.115	1.49	0.91	2.45	0.115
Antidiarrhoeal	3.52	0.79	15.7	0.099	3.38	0.75	15.21	0.112
Harmful (antibiotic or antidiarrhoeal)	1.71	1.1	2.76	**0.029***	1.72	1.1	2.82	**0.030***
Not recommended	0.84	0.49	1.45	0.54	0.87	0.5	1.52	0.629
Schedule H	1.62	1.01	2.61	**0.046***	1.62	1	2.65	**0.052***
Schedule H1	1†				1			
Referral	0.85	0.53	1.37	0.507	0.82	0.51	1.34	0.431

*significant at 5% level

† standard error omitted (very few observations)

Table A4: Impact of pharmacy type on therapeutic management of suspected TB case, with and without SP fixed-effects

Variable	Unadjusted Odds Ratio	95% Lower Bound	95% Upper Bound	P-value	Adjusted Odds Ratio	95% Lower Bound	95% Upper Bound	P-value
**Correct case management**	1.09	0.68	1.74	0.726	1.06	0.65	1.73	0.822
**Referral**	1.12	0.7	1.79	0.632	1.1	0.67	1.8	0.711
**Antibiotic**	0.97	0.52	1.82	0.924	1.03	0.54	1.95	0.933
**Steroid**	3.2	0.39	26.37	0.279	3.36	0.41	27.8	0.261
**Harmful (antibiotic or steroid)**	1	0.54	1.87	0.997	1.06	0.56	2.01	0.852
**Not recommended**	0.96	0.59	1.55	0.871	0.96	0.58	1.59	0.882
**Schedule H**	0.91	0.57	1.45	0.685	0.93	0.58	1.51	0.782
**Schedule H1**	1†							
**Anti-tuberculosis medicine**	n/a							

† standard error omitted (very few observations)
